# Diversity of avipoxviruses in captive-bred Houbara bustard

**DOI:** 10.1186/s13567-014-0098-3

**Published:** 2014-10-01

**Authors:** Guillaume Le Loc’h, Mariette F Ducatez, Christelle Camus-Bouclainville, Jean-Luc Guérin, Stéphane Bertagnoli

**Affiliations:** RENECO Wildlife Consultants LLC, Abu Dhabi, United Arab Emirates; INRA, UMR1225, IHAP, F-31076 Toulouse, France; Université de Toulouse, INP, ENVT, UMR1225, IHAP, F-31076 Toulouse, France

## Abstract

Implementation of conservation breeding programs is a key step to ensuring the sustainability of many endangered species. Infectious diseases can be serious threats for the success of such initiatives especially since knowledge on pathogens affecting those species is usually scarce. Houbara bustard species (*Chlamydotis undulata* and *Chlamydotis macqueenii*), whose populations have declined over the last decades, have been captive-bred for conservation purposes for more than 15 years. Avipoxviruses are of the highest concern for these species in captivity. Pox lesions were collected from breeding projects in North Africa, the Middle East and Central Asia for 6 years in order to study the diversity of avipoxviruses responsible for clinical infections in Houbara bustard. Molecular and phylogenetic analyses of 113 and 75 DNA sequences for P4b and fpv140 loci respectively, revealed an unexpected wide diversity of viruses affecting Houbara bustard even at a project scale: 17 genotypes equally distributed between fowlpox virus-like and canarypox virus-like have been identified in the present study. This suggests multiple and repeated introductions of virus and questions host specificity and control strategy of avipoxviruses. We also show that the observed high virus burden and co-evolution of diverse avipoxvirus strains at endemic levels may be responsible for the emergence of novel recombinant strains.

## Introduction

Avipoxviruses (APV) are large enveloped, double stranded DNA viruses able to naturally infect more than 232 species from 23 orders [1]. They belong to the genus *Avipoxvirus*, subfamily *Chordopoxvirinae* and family *Poxviridae*. To date, 10 APV species have been defined by the International Committee on Taxonomy of Viruses [2]. Phylogenetic analyses of APV have previously been based on a couple of genes. An amplification product of 578 bp from the *fpv167* gene, orthologous of vaccinia virus A3L gene encoding a virion core protein 4b, has been widely used since this gene is highly conserved among poxviruses [3]. Other genes have been used in order to validate or improve findings based on *fpv167*. The fpv140 locus (*fpv139*, *fpv140* and *fpv141* genes), has also been used [4] and the phylogenetic analyses based on *fpv140*, orthologous of vaccinia virus H3L gene encoding a virion envelope protein p35, have provided improved strain discrimination within some subclades [5-7]. Phylogenetic analyses have all shown the same distribution of APV into 3 clades: fowlpox virus-like (FWPV-like, clade A), canarypox virus-like (CNPV-like, clade B), and psittacinepox virus-like (clade C).

APV infections can cause significant economic losses in domestic poultry, due to decreased egg production, reduced growth, blindness and increased mortality [8]. In wild birds, the infection can compromise survival and breeding success by decreasing the ability to escape predators [9], to fledge and rear chicks [10], by impairing the pairing success [11] or by increasing mortality [12]. Recently, emergence of APV infections in great tits in Europe and especially in the UK has been described and could represent a threat for the species [13,14]. The impact of the disease can be dramatic for endangered species, especially for island species when APV are introduced in naïve populations as described in Hawaii [15], the Galapagos Islands [16] or Canary islands [17]. The disease is also a concern for the success of conservation programs of endangered species, such as peregrine falcons in Germany [18] or Houbara bustard species (hereafter “Houbara”) in the United Arab Emirates (UAE) and Morocco [19,20].

The African Houbara bustard (*Chlamydotis undulata*) and the Asian Houbara bustard (*Chlamydotis macqueenii*) inhabit semi-arid areas in North Africa, the Middle East and Central Asia. In the last decades, their populations drastically declined due to over-hunting, habitat degradation and poaching [21] leading the species to be listed as “Vulnerable” by the International Union for Conservation of Nature (IUCN 2012). For more than 15 years, captive-breeding programs of the Houbara have been implemented in North Africa, the Middle East and Central Asia. Their goal is to increase the size of wild populations through release of captive-born individuals, which should substantially decrease extinction risk. APV infections are known to compromise the success of Houbara captive-breeding programs by increasing mortality [19,20]. Persistent presence of infections is observed in captive flocks with morbidity rates of 2-3% in juvenile birds (≤1 year) despite prophylactic programs (G. Le Loc’h, unpublished data) but very little information is available about the APV strains circulating in Houbara.

The present study was aimed at assessing APV diversity within a species as well as virus circulation within captive-breeding programs. Molecular and phylogenetic analyses of APV in Houbara highlight that a large pathogen diversity and burden is possible in an apparent unfavorable environment.

## Materials and methods

### Animal samples

The study was conducted from 2008 through 2013 in 3 Houbara captive-breeding projects: the Emirates Center for Wildlife Propagation in Morocco, the National Avian Research Center and the Sheikh Khalifa Houbara Breeding Center in the UAE and the Emirates Centre for the Conservation of Houbara in Uzbekistan. Birds housed in these projects were either *C. macqueenii* alone (UAE and Uzbekistan) or *C. macqueenii* and *C. undulata* (Morocco). Pox lesions were detected during routine check-up as part of veterinary management of birds. When typical lesions of APV infections, either cutaneous (nodular lesions on nonfeathered areas) or diphtheritic (yellowish lesions on the mucous membranes of the mouth, esophagus, or trachea), were observed [8], they were aseptically removed, transferred into a sterile pot and stored at −80 °C until analysis. In total, 169 lesions were sampled (Table 1): 52 in *C. undulata* and 117 in *C. macqueenii*, mainly on juvenile birds (≤ 1 year; *n* = 143, 85%).

**Table 1:** **Distribution of lesions collected in Houbara bustard captive-breeding projects from 2008 through 2013**

	**Morocco**	**United Arab Emirates**	**Uzbekistan**
2008	7	-	-
2009	6	-	-
2010	21	1	-
2011	49	4	-
2012	11	7	12
2013	6	45	-
	100	57	12

DNA was extracted from 25 mg of frozen tissue samples with the NucleoSpin Tissue kit (Macherey-Nagel, Düren, Germany) according to the manufacturer’s instructions.

### PCR, electrophoresis, and sequencing

PCR primers previously described were used to amplify P4b [22] and fpv140 loci [4]. PCR were performed with the Illustra puReTaq Ready-To-Go PCR Beads kit (GE Healthcare, Little Chalfont, UK) in a Mastercycler gradient thermocycler (Eppendorf, Hamburg, Germany). Reaction volume was 25 μL and contained 3 μL of the target DNA, 1 μL of each primer (10 μmol/L), 20 μL of water and 1 re-solubilized bead (DNA polymerase, dNTP and reaction buffer). Cycling conditions for P4b amplification consisted of an initial denaturation for 5 min at 95 °C followed by 35 cycles of denaturation (95 °C, 40 s), annealing (60 °C, 40 s) and extension (72 °C, 1 min), and ended by a final extension for 10 min at 72 °C. For amplification of fpv140, an initial denaturation (95 °C, 5 min) was followed by 35 cycles of denaturation (95 °C, 40 s), annealing (52 °C, 40 s) and extension (72 °C, 2 min), and ended by a final extension for 10 min at 72 °C.

PCR amplicons (5 μL) were analyzed on 1.2% (P4b) or 1% (fpv140) agarose gels with TBE and SYBR Safe DNA Gel Stain (Invitrogen, Carlsbad, USA). PCR products were purified using NucleoSpin Gel and PCR Clean-up kit (Macherey-Nagel), eluted in 30 μL of water and subsequently sequenced using the amplification primers and internal primers on an ABI 3130 XL automated DNA sequencer (Applied Biosystems, Foster City, USA) at the Plateau de Génomique GeT-Purpan, UDEAR UMR 5165 CNRS/UPS, CHU PURPAN, Toulouse, France. In order to extend the reads of fpv140 sequences, the following internal primers were used: FPV140_01F (5′-ATCCTGCGGCTGAACAGTAT-3′), FPV140_02F (5′-TTATCCTAGATTTTATGGATGATTTTG-3′), FPV140_02R (5′-TTCTGCTAAGTTGCCGGAAT-3′), FPV140_03F (5′GACGACATCATTCTGATTTCCTTA-3′), FPV140_03R (5′-AAAAATTCTATCGCCAATCACA-3′), FPV140_04R (5′-AACACATACCAAATTGCTAAAAGA-3′), FPV140_06F (5′-TGTACACATTTATCCATAAACTCTCCT-3′), FPV140_08F (5′-GAATAGCAGTATCCAGATTCGCT-3′), FPV140_08R (5′-CTGTTGCAAGAGACGGCTTT-3′), FPV140-09R (5′-GCTAACCATGTGAGTCTGTGG-3′) and FPV140_10R (5′-TGCAGGCTTACATGTACAGAAG-3′).

### Phylogenetic and recombination analyses

After manual editing and excluding primers with BioEdit 7.2.5, P4b and fpv140, DNA sequences were aligned with sequences available on GenBank using ClustalX 2.1. All available sequences on GenBank were initially used, then, one unique sequence of each group was kept for phylogenetic analyses. APV strains collected during the present study were named with the following nomenclature: APV clade (as determined by phylogenetic analysis of P4b: FWPV, CNPV and PSPV)/host species/origin/strain number/collection year. For example, CNPV/*Chlamydotis macqueenii*/AE/072/2011 is a Canarypox virus-like strain number 072 collected from a *Chlamydotis macqueenii* in the UAE in 2011. GenBank accession numbers are available in Table 2. Phylogenetic trees were generated using MEGA 5.2.1 by neighbor-joining (NJ) with the Kimura 2-parameters model and reliability of trees was tested through 1000 bootstrap replicates. After identification of clades and subclades, within and between subclades mean genetic distances were calculated with the same algorithm.

**Table 2:** **GenBank accession numbers of strains isolated and used in this study**

**Strains**	**GenBank accession numbers**	
	**P4b locus**	**fpv140 locus**
CNPV/*Chlamydotis macqueenii*/AE/072/2011	LK021648	LK021667
CNPV/*Chlamydotis macqueenii*/MA/006/2011	LK021649	LK021668
CNPV/*Chlamydotis macqueenii*/MA/012/2010	LK021650	LK021669
CNPV/*Chlamydotis macqueenii*/MA/023/2011	LK021651	LK021670
CNPV/*Chlamydotis macqueenii*/MA/031/2010	LK021652	LK021671
CNPV/*Chlamydotis macqueenii*/MA/036/2010	LK021653	LK021672
CNPV/*Chlamydotis macqueenii*/MA/083/2011	LK021654	LK021673
CNPV/*Chlamydotis macqueenii*/MA/097/2012	LK021655	LK021674
CNPV/*Chlamydotis macqueenii*/UZ/102/2012	LK021656	LK021675
CNPV/*Chlamydotis macqueenii*/UZ/103/2012	LK021657	LK021676
CNPV/*Chlamydotis undulata*/MA/001/2009	LK021658	LK021677
CNPV/*Chlamydotis undulata*/MA/010/2010	LK021659	LK021678
CNPV/*Chlamydotis undulata*/MA/025/2011	LK021660	LK021679
CNPV/*Chlamydotis undulata*/MA/077/2009	LK021661	LK021680
FWPV/*Chlamydotis macqueenii*/AE/066/2012	LK021662	LK021681
FWPV/*Chlamydotis macqueenii*/AE/070/2011	LK021663	LK021682
FWPV/*Chlamydotis macqueenii*/AE/119/2013	LK021664	LK021683
FWPV/*Chlamydotis macqueenii*/AE/150/2013	LK021665	LK021684
FWPV/*Chlamydotis undulata*/MA/024/2011	LK021666	LK021685

A recombination analysis was performed with RDP v3.44 in order to detect potential recombination events for CNPV/*Chlamydotis undulata*/MA/001/2009, which grouped differently when analyzing P4b and *fpv140*. Concatenated sequences of fpv140 (2750 to 2878 bp) and P4b (487 to 490 bp) loci were treated as linear by RDP, GeneConv, BootScan, MaxChi, Chimaeara, SiScan and 3Seq methods. Settings were adjusted to detect events with a highest acceptable p value equal to 0.0001.

In order to exclude a mix of different sequences in CNPV/*Chlamydotis undulata*/MA/001/2009, which could explain differences of positioning in phylogenetic trees, primers specific to either B1 (FPV140_03F and FPV140_03R) or B2 subclades (FPV140_02F, and FPV140_02R), and targeting the fpv140 locus were used. PCR were performed on CNPV/*Chlamydotis undulata*/MA/001/2009 and one additional specimen of subclades B1 and B2 were used as positive controls.

## Results

### Phylogeny

Out of the 169 samples, 139 DNA sequences were successfully amplified with at least one set of primers. Discrimination between FWPV-like viruses and CNPV-like viruses based on fpv140 loci fragment size shows a slightly higher proportion of CNPV-like viruses (*n* = 63, 55%) than FWPV-like viruses (*n* = 51, 45%).

Thereafter, 113 and 75 DNA sequences were obtained for P4b and fpv140, respectively. P4b sequences were trimmed to a 426 bp length fragment, which was used to carry out phylogenetic analysis. From fpv140 sequences, the 3 open reading frames (ORF) corresponding to the 3 orthologous genes shared by FWPV-like viruses and CNPV-like viruses (*fpv139*/CNPV184, *fpv140*/CNPV186 and *fpv141*/CNPV187) were used to build independent phylogenetic trees.

**Figure 1:**
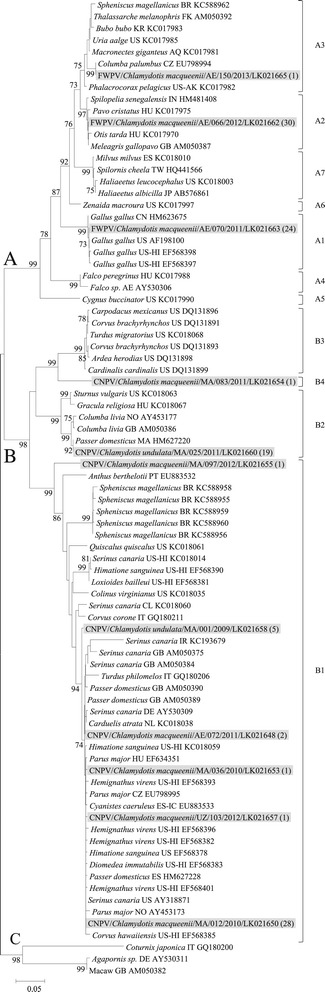
**Neighbor-joining phylogenetic tree of P4b DNA sequences.** The neighbor-joining phylogenetic tree was constructed with the Kimura 2-parameters model. Bootstrap values (1000 replicates) > 70% are shown. The 113 sequences identified in the present study grouped in 11 genotypes represented by strains are highlighted in grey. The numbers in brackets correspond to the numbers of strains identified in the present study, which have an identical P4b DNA sequence of 426 bp. Sequences obtained from GenBank are named as follows: host species/origin/accession number. Strains subclades are indicated on the right hand side of the tree.

The NJ tree based on P4b DNA sequences (Figure 1) provided clear distinction between known APV clades and subclades as previously described [4,23]. The strains identified in the present study clustered in 11 genotypes belonging to clades A and B. Most of the strains of clade A grouped either in subclade A2 (*n* = 30, genotype represented by FWPV/*Chlamydotis macqueenii*/AE/066/2012) with 100% identity with sequences obtained from a wide diversity of wild and domestic species, or in subclade A1 (*n* = 24, genotype represented by FWPV/*Chlamydotis macqueenii*/AE/070/2011) with 100% identity with sequences from domestic Galliformes (e.g. *Gallus gallus*). FWPV/*Chlamydotis macqueenii*/AE/150/2013 grouped in subclade A3 and was identical to sequences from Columbiformes (e.g. *Columba palumbus*) and a great bustard (*Otis tarda*) in Spain. The 8 genotypes of clade B grouped in 3 subclades: the previously described subclades B1 (*n* = 38) and B2 (*n* = 19), and a putative new subclade B4 (*n* = 1). Six genotypes grouped in subclade B1, which comprised sequences from a wide diversity of species, mainly Passeriformes. The predominating genotype (*n* = 28, represented by CNPV/*Chlamydotis macqueenii*/MA/012/2010) shared 100% identity with sequences from great tits (*Parus major*) in Europe and house sparrows (*Passer domesticus*) in Morocco. The genotype represented by CNPV/*Chlamydotis macqueenii*/AE/072/2011 (*n* = 2) was identical to a sequence from an African Houbara in the UAE. Only one genotype (*n* = 19, represented by CNPV/*Chlamydotis undulata*/MA/025/2011) grouped in subclade B2 was identical to sequences from house sparrows in Morocco. Phylogenetic analysis shows a putative new subclade, B4, which comprised only one strain from an Asian Houbara in Morocco, CNPV/*Chlamydotis macqueenii*/MA/083/2011 (Figure 1). The closest sequence (90% identity) was isolated from an American robin (*Turdus migratorius*) in the United States and clustered in subclade B3. The minimal genetic distance between subclade B4 and other B subclades was 10.8% (Table 3).

**Table 3:** **Within and between subclade mean genetic distances for P4b sequences**

	**Between subclade distances (%)**											**Within subclade distances (%)**
	**A1**	**A2**	**A3**	**A4**	**A5**	**A6**	**A7**	**B1**	**B2**	**B3**	**B4**	
A1												0.3
A2	10.3											0.6
A3	9.3	3.2										1.4
A4	12.3	11.7	12.4									1.2
A5	14.8	13.7	12.3	16.3								-
A6	8.9	5.9	4.9	11.4	11.4							-
A7	10.5	6.7	5.2	13.7	11.9	6.1						0.7
B1	29.6	30.1	31.1	28.7	27.5	28.6	31.3					4.8
B2	27.6	25.7	25.5	24.3	28.2	25	28.6	17.9				2.7
B3	27.9	25.4	25.4	26.9	25.1	24	25.3	20.4	15.8			0.6
B4	28.2	26.2	26.3	25.7	27.5	26.5	29.1	21.1	15.4	10.8		-
C	30.4	32.3	32.1	33.5	31.9	29.8	32.2	32.5	30.8	34.5	31.1	19.4

Distances were calculated by neighbor-joining with the Kimura 2-parameter model.

**Figure 2:**
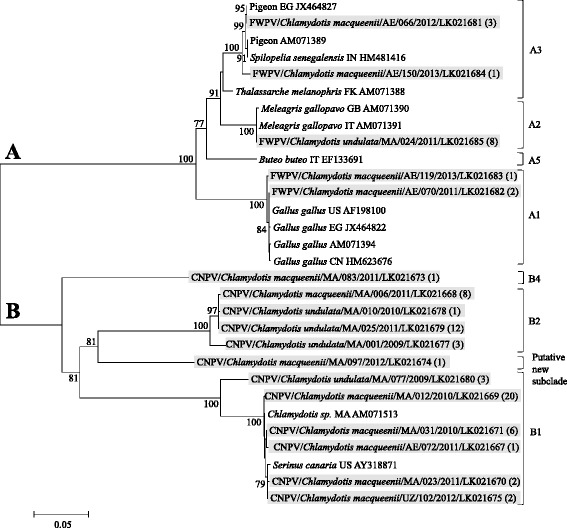
**Neighbor-joining phylogenetic tree of fpv140 DNA sequences.** Neighbor-joining phylogenetic tree was constructed with the Kimura 2-parameters model. Bootstrap values (1000 replicates) > 70% are shown. The 75 sequences identified in the present study grouped in 17 genotypes are represented by strains highlighted in grey. The numbers in brackets correspond to the numbers of strains identified in the present study, which have an identical *fpv140* DNA sequence. Sequences obtained from GenBank are named as follows: host species/origin/accession number. Strains subclades are indicated on the right hand side of the tree.

The complete *fpv140* gene sequence (933–1020 bp) was used to build an NJ tree (Figure 2). Its analysis provided the clades/subclades classification as previously described for clade A [4,5] and new information for clade B. The 75 strains identified in the present study clustered in 17 genotypes, 5 belonging to clade A and 12 belonging to clade B. Among strains which grouped in subclade A2 as per phylogeny based on P4b (FWPV/*Chlamydotis macqueenii*/AE/066/2012), some remained in subclade A2 (sequences from domestic turkeys – *Meleagris gallopavo*), and others grouped in subclade A3 (mainly sequences from Columbiformes). In clade B, CNPV/*Chlamydotis undulata*/MA/001/2009 and CNPV/*Chlamydotis macqueenii*/MA/097/2012, which grouped in subclade B1 with P4b, were positioned differently. CNPV/*Chlamydotis undulata*/MA/001/2009 grouped in subclade B2 while CNPV/*Chlamydotis macqueenii*/MA/097/2012, which did not share more than 76.2% identity with other sequences, formed a putative new subclade. CNPV/*Chlamydotis macqueenii*/MA/083/2011 grouped in a putative new subclade B4 as identified with the phylogeny based on the P4b gene (Figure 2).

The analysis of the NJ trees built with *fpv139* and *fpv141* DNA sequences (data not shown) provided essentially the same information as obtained with *fpv140*.

### Identification of recombination events

**Figure 3:**
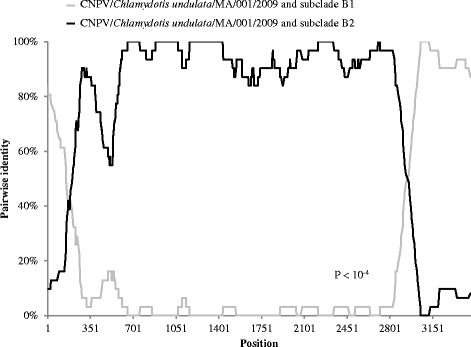
**Evidence of recombination between CNPV/Chlamydotis undulata/MA/001/2009 and subclades B1 and B2 strains.** Evidence of recombination was calculated by the RDP method from concatenated sequences of fpv140 and P4b loci and with *p* value < 0.0001. The percent nucleotide similarity between CNPV/*Chlamydotis undulata*/MA/001/2009 and subclade B1 (represented by CNPV/*Chlamydotis macqueenii*/MA/012/2010) and between CNPV/*Chlamydotis undulata*/MA/001/2009 and subclade B2 (represented by CNPV/*Chlamydotis undulata*/MA/025/2011) is represented by a grey and a black line, respectively.

Recombination analysis was performed from concatenated DNA sequences of fpv140 and P4b loci from CNPV/*Chlamydotis undulata*/MA/001/2009, CNPV/*Chlamydotis macqueenii*/MA/012/2010 (subclade B1) and CNPV/*Chlamydotis undulata*/MA/025/2011 (subclade B2). Concatenated sequence sizes ranged from 3255 to 3386 bp. The 7 methods used show evidence of recombination between genotypes represented by CNPV/*Chlamydotis macqueenii*/MA/012/2010 and CNPV/*Chlamydotis undulata*/MA/025/2011, with a significant *p* value (*p* < 0.0001, Figure 3). The recombination points were positioned intra-locus at position 208 of the fpv140 locus (in *fpv139* gene) and inter-loci: between the fpv140 and P4b loci. CNPV/*Chlamydotis undulata*/MA/001/2009 was closer to CNPV/*Chlamydotis undulata*/MA/025/2011 between those 2 points while it was closer to CNPV/*Chlamydotis macqueenii*/MA/012/2010 outside of those points.

**Figure 4:**
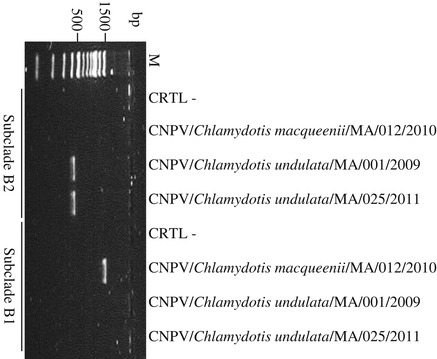
**PCR amplification of an internal segment of the fpv140 locus for discrimination between subclades B1 and B2.** Lane M, 100 pb DNA size marker; CRTL-, negative control. CNPV/*Chlamydotis macqueenii*/MA/012/2010 and CNPV/*Chlamydotis undulata*/MA/025/2011 cluster in subclades B1 and B2, respectively. Subclades B1 and B2 amplification product sizes: 1526 and 448 bp, respectively.

To exclude a mix of sequences in CNPV/*Chlamydotis undulata*/MA/001/2009, a PCR was carried out on its DNA showing an amplification product with subclade B2 specific primers but no amplification product with subclade B1 specific primers (Figure 4).

### Geographic distribution

**Figure 5:**
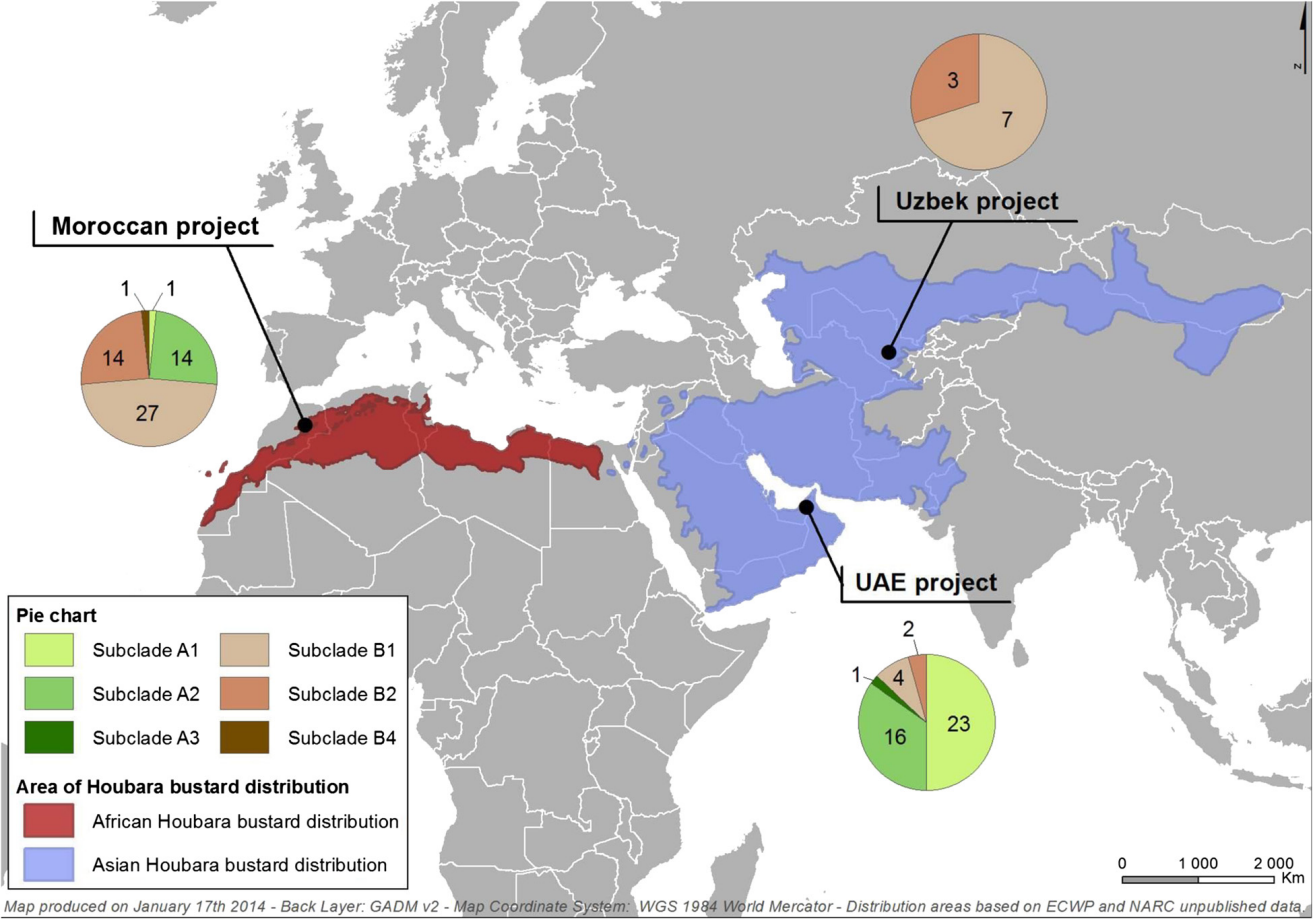
**Distribution of strains collected in Houbara bustards in 3 breeding projects.** The 3 breeding projects cover the natural distribution of African Houbara bustard (*Chlamydotis undulata*) and Asian Houbara bustard (*Chlamydotis macqueenii*). The numbers in pie charts correspond to the number of strains grouping in P4b subclades.

Out of the 113 P4b sequences obtained in the present study, 57 were collected in Morocco, 46 in the UAE and 10 in Uzbekistan (Figure 5). Sequences of several subclades were obtained on each site with differences among projects. CNPV-like viruses were predominant in Morocco with 74% of the strains grouping in clade B (47% and 25% in subclades B1 and B2, respectively). FWPV-like viruses were mainly represented by strains of subclade A2 (25%). In the UAE, 87% of the strains were FWPV-like viruses, grouping in subclades A1 and A2 for 50% and 35% of the viruses, respectively. Only CNPV-like viruses were identified in Uzbekistan with 70% of the strains grouping in subclade B1. Strains of subclades A3 and B4 were identified in only one site, the UAE and Morocco, respectively.

## Discussion

APV infections in Houbara have been poorly studied and only 2 DNA sequences from these species are published in GenBank. This study contributes to a better comprehension of the disease in Houbara by providing 113 sequences for P4b and 75 sequences for fpv140, collected over 6 years in 3 different countries, which cover the natural distribution of Houbara. Virus isolates have been obtained for a few representative strains (FWPV and CNVP-like viruses: at least 1 isolate per subclade, data not shown).

The NJ phylogenetic tree built from P4b DNA sequences was very similar to those already published [4,5,23] for clade A and distinguished 7 subclades, while CNVP-like sequences from Houbara provided more details in clade B. The use of a second locus gave a better resolution within and between some subclades. The fpv140 locus encompasses an entire ORF (*fpv140* gene) ranging from 933 to 1020 bp and despite reports of difficulties to amplify the gene [24], its amplification was successful for 84% of Houbara strains for which a P4b gene sequence had been obtained. The number of APV sequences available has more than doubled thanks to the 75 sequences generated here.

A putative new subclade (B4) was identified from an Asian Houbara in Morocco in 2011 (CNPV/*Chlamydotis macqueenii*/MA/083/2011). Its minimal genetic distance with other B subclades (10.8%) was higher than some between A subclade genetic distances (3.2% to 16.3%, Table 3), which justified considering it as a new subclade. CNPV/*Chlamydotis macqueenii*/MA/083/2011 seemed to share the same ancestor as subclade B3 isolates, all originating from the USA.

Subclade B1, whose diversity is the highest as compared with other subclades, is usually subdivided into 2 clusters [25]. More recently, a third cluster was described for APV isolates from Magellanic penguins [26]. With Houbara sequences, especially CNPV/*Chlamydotis undulata*/MA/001/2009 and CNPV/*Chlamydotis macqueenii*/MA/097/2012, diversity within subclade B1 increased and distinction of clusters becomes questionable. Also, it questions the definition of this group of virus as a unique subclade since the intra-B1 genetic distance (4.8%) is higher than the genetic distance between subclades A2 and A3 (3.2%).

This is the first time that so many sequences are isolated from a single species (*n* = 84 for *C. macqueenii*; *n* = 29 for *C. undulata*), thus providing a better understanding of the epidemiology of APV for each species. The diversity of APV isolated in Houbara is surprising. Published sequences from Houbara suggested that they are usually infected by CNPV-like viruses [27]. In this study, we show that Houbara viruses grouped in 11 different genotypes and 6 different subclades when looking at P4b (17 genotypes, 7 subclades with *fpv140*) and almost half of them grouped with FWPV-like viruses. Classically, APV are considered to be host species or order specific and taxonomy was based on this concept. This has been recently questioned by many authors [4-6,23] since some taxa like *Columbidae* and *Accipitridae* can be infected by a wide diversity of strains, however with different sensitivity depending on the virus involved. This apparent diversity could be explained by some infections occurring as accidental events, especially in zoological collections where many species are housed closely, and it is suggested that such an infection could not lead to sustainable epornitics [23]. However, in the present study, all genotypes identified were responsible for clinical infections in Houbara and lead to nodular lesions on legs, beaks or eyelids without any evidence of phenotype-genotype relationship. CNPV-like viruses as well as FWPV-like viruses were also identified from diphtheritic lesions and could be responsible for outbreaks in Houbara captive-breeding projects as observed in Morocco in 2009 or in the UAE in 2013 with outbreaks of CNPV-like viruses and FWPV-like viruses respectively (G. Le Loc’h, unpublished data). This suggests that the taxonomy of APV may be reviewed and questions host specificity of APV.

The wide diversity of strains affecting Houbara has also been observed within a single breeding project and for a single year since strains of 5 clades were collected in Morocco in 2011 and strains of 4 clades were collected in the UAE in 2013. Previous studies have shown little diversity of strains in geographic areas of the same scale, within one species [26] as well as among different species [28]. This has been explained either by the quick spread of a competent virus in immunologically naïve birds [28] or by a limited number of introductions of strains in insular populations [25,29,30]. The high diversity observed in this study within a single breeding station suggests that multiple and repeated introductions of virus occurred. The origin of introductions can be hypothesized through observation of phylogenetic trees. The gene *fpv140* provides a better resolution than the P4b locus for FWP-like viruses since each subclade used to be linked to a host species or order. Moreover the advantage of using *fpv140* for virus typing could be attributable to the surface nature of the encoded H3L orthologue ORF. Subclade A1 viruses were exclusively collected from domestic chickens and Houbara strains clustering in this subclade all originated from the UAE where poultry farms surround breeding stations. The same observation can be made for subclade A3 in which most strains were collected from Columbiformes. Most of the Houbara strains grouping in subclade A3 originated from the UAE where large populations of doves and pigeons live around the breeding stations. On the contrary, subclade A2 viruses were all collected from domestic turkeys and subclade A2 Houbara strains originated mainly from Morocco, where backyard turkeys are found in villages surrounding breeding stations. Origins of CNPV-like viruses collected in this study are more difficult to precise since the host-subclade relationship in clade B is less evident. However some viruses collected from Houbara in Morocco were identical for P4b to viruses originating from Moroccan house sparrows in subclade B1 as well as in subclade B2.

The multiple origin of viruses affecting Houbara questions the role of the species in the APV epidemiological cycle. It could be assumed that this species is especially sensitive to APV viruses regardless of their origin. Management of captive-breeding projects can also provide some clues to understanding our observation. Most of the rearing process takes place in outdoor aviaries [31], where the birds are housed in small groups, allowing potential contact with wild birds and insect vectors. Moreover, although projects are located in quite hostile and isolated landscapes (semi-desert or desert), they are usually built with all accommodations needed to ensure a certain autonomy (alfalfa fields to feed the Houbara, gardens for staff, etc.). Those artificial oases are especially attractive for wild fauna and can concentrate relatively large populations of wild birds and insects. In addition, some projects such as the ones in Morocco are located along migratory pathways. In contrast, due to a high conservation value of each Houbara and despite flock size of thousands of birds in each project, every bird is managed individually and carefully controlled with a high biosecurity level. While this management prevents quick and massive spread of pathogens, it may allow a higher diversity of virus strains entering and co-evolving at an endemic level in the breeding stations by breaking the epidemiological cycle of APV.

This observation questions the prophylactic strategy to control APV infection. Sensitivity of Houbara, as other birds’ species, to different APV strains appears higher than expected. Frequent viral challenge even in an apparent unfavorable environment, as shown by the multiple introductions of APV in Houbara breeding stations, is also surprising. This makes the association of different prophylactic measures such as classical biosecurity, prevention of wild bird concentration, control of insect vector populations and vaccination, necessary. Vaccination alone is questionable regarding the wide diversity of strains affecting Houbara and the poorly known cross-protection among APV. Previous studies give contradictory conclusions about APV cross-immunity. While most of them show no cross-protection between clades [32,33] and even inside the same clade [34], a more recent study provides evidence of cross-protection between FWPV-like viruses and CNPV-like viruses in experimentally infected zebra finches [35]. In FWPV-like viruses, the absence of cross-protection has been shown to be linked to the presence of the integrated reticuloendotheliosis virus (REV) sequence in the FWPV genome [34]. In Houbara strains, REV was only detected in the FWPV subclade A1 viruses (data not shown). The latter studies suggest that cross-protection could be related to APV strains but also to host species. Further investigations are warranted to improve our understanding of the determinism of host specificity and pathogenicity among different APV strains.

The presence of several different strains within the same population at the same moment is favorable to the emergence of recombinant strains as already shown in orthopoxviruses [36] and is suspected for APV [23]. In Morocco, strains belonging to subclades B1 and B2 as well as a potential recombinant strain were isolated within the same breeding station in November 2009 and again in July 2011 (G. Le Loc’h, unpublished data), highlighting the high viral load in the field. The analyses confirmed the hypothesis of recombination observed on phylogenetic trees by showing intra and inter-loci recombination events. Interestingly, the parent strains from both subclades B1 and B2 (group of isolates CNPV/*Chlamydotis macqueenii*/MA/012/2010 and CNPV/*Chlamydotis undulata*/MA/025/2011, respectively) are the only ones isolated in the 3 Houbara breeding projects and their P4b sequences are identical to those of strains isolated from house sparrows in Morocco [37]. Moreover, the P4b sequence of the subclade B1 parent is identical to a strain distributed worldwide and that has been more especially isolated from great tits in Europe and associated with the emergence of APV infections in this species [13,14]. It can be hypothesized that this is a unique B1 strain that may have the ability to effectively and sustainably infect a wide diversity of avian hosts. However, whole genome sequences would be necessary to conclude on the unicity of the strain. Understanding APV evolution requires better knowledge of viral genomes. Up to now, the genomes of only 4 APV species have been fully sequenced: those of *Fowlpox virus* [38], *Pigeonpox virus*, *Penguinpox virus* [39], and *Canarypox virus* [40], 288 kbp and 365 kbp long, respectively. Their analyses have shown large genomic rearrangements and suggest significant genomic diversity among APV. Effort should be made to obtain complete genome sequences of several APV in order to better understand their host adaptability and their pathogenicity.
